# A novel method of combining generalized frequency response function and convolutional neural network for complex system fault diagnosis

**DOI:** 10.1371/journal.pone.0228324

**Published:** 2020-02-04

**Authors:** Lerui Chen, Zerui Zhang, Jianfu Cao

**Affiliations:** State Key Laboratory for Manufacturing Systems Engineering, Xi'an JiaoTong University, Xi’an, China; Newcastle University, UNITED KINGDOM

## Abstract

To solve the problem of low accuracy in traditional fault diagnosis methods, a novel method of combining generalized frequency response function(GFRF) and convolutional neural network(CNN) is proposed. In order to accurately characterize system state information, this paper proposed a variable step size least mean square (VSSLMS) adaptive algorithm to calculate the second-order GFRF spectrum values under normal and fault states; In order to improve the ability of fault feature extraction, a convolution neural network (CNN) with gradient descent learning rate and alternate convolution layer and pooling layer is designed to extract the fault features from GFRF spectrum. In the proposed method, the second-order GFRF spectrum of each state of Permanent Magnet Synchronous Motor (PMSM) is obtained by VSSLMS; Then, the two-dimension GFRF spectrum, which is regarded as the gray value of the image,will be further transformed into image. Finally, the CNN is trained with learning rate by gradient descent way to realize the fault diagnosis of PMSM. Experimental results indicate that the accuracy of proposed method is 98.75%, which verifies the reliability of the proposed method in application of PMSM fault diagnosis.

## 1 Introduction

Permanent magnet synchronous motor (PMSM) is widely used in industrial robot drive system because of its small size, light weight and simple structure. Because PMSM works in continuous and heavy load environment, it is easy to suffer from abnormal fever, rotor poor lubrication and rotor magnetic leakage[1], so it is very important to monitor and diagnose the fault of PMSM.

At present, with the deepening research of motor, there are many researches on motor fault diagnosis. Most of them are based on monitoring and acquisition of motor state-related signal, such as voltage, speed, current and vibration signal, and then using feature extraction, information fusion and pattern recognition methods to achieve fault diagnosis.Ref. [2] considered stator current as fault signal feature to detect the fault of planetary gearbox of motor at different rotational speeds; Ref. [3] proposed a feature extraction method of smofs-22,which based on acoustic signal to diagnose stator, rotor and bearing faults of single-phase induction motor; Ref. [4] treated current signal as fault features, and convert the current signal by continuous wavelet transform (CWT) to realize the diagnosis of broken rotor bars in squirrel cage induction motor; Ref. [5] utilized the vibration signal in frequency domain to realize the motor bearing fault diagnosis by one-dimension fusion neural network; Ref. [6] applied discrete wavelet transform (DWT) to the stator current signal to realize the fault diagnosis of motor rotor. These methods above only analyzed single signal, such as current or vibration signal, which is difficult to extract fault characteristics effectively; Ref.[7] proposed a multi-source health state information fusion method based on DS theory, which can achieve better information fusion for automobile motor from X-axis, Y-axis and Z-axis and get good prediction results of health status; Ref. [8] proposed a fault signal classification method based on support vector machine (SVM) and short-time Fourier transform (STFT) for sensor data fusion, which combined vibration, current, voltage, sound and temperature information to detect and identify motor faults; Ref. [9] fused the vibration signal collected from two-direction of the motor, then the fused vibration signal is decomposed into multiple scales by continuous wavelet transform (CWT), and the first scale is used to represent the degradation of bearing. Although these methods above make up for the limitation of single signal by fusing two or more signal, the fault features were extracted from the output signal of the system without considering the influence of the input signal on the system, so it cannot fully reflect the non-linear characteristics of the system. Nonlinear spectrum analysis based on generalized frequency response function (GFRF) considers the influence of input on the system characteristics and can characterize the dynamic characteristics of the nonlinear system. Nonlinear frequency spectrum can well explain the characteristics of system, the accuracy of fault diagnosis can be improved by using it to characterize the state information, and achievements have been made in the field of fault diagnosis which can be shown in Refs. [10]-[15].However, up to now, it is very rare to study the fault diagnosis for permanent magnet synchronous motor (PMSM) based on GFRF spectrum.

In recent years, with the increasing number of equipment monitoring system, the monitoring system has obtained a large number of data, so the fault diagnosis method based on data-driven has been favored by scholars. Among many data-driven methods, deep learning has advantages in image classification with its powerful learning and recognition ability, and also has been applied in the field of fault diagnosis; Ref.[16] proposed a motor fault diagnosis method based on stacked de-noising auto-encoder, which can extract features adaptively. Compared with traditional learning methods, it can extract fault features more deeply and obtain higher diagnosis accuracy; Ref. [17] proposed deep belief networks (DBN), which can learn the characteristics of vibration signal from the frequency distribution to characterize the working state of induction motors. Combining feature extraction and classification, the intelligent fault diagnosis is realized; Ref.[18] combined stacked de-noising auto encoders (SDAE) and nonlinear output frequency response function(NOFRF) spectrum to realize the fault diagnosis of permanent magnet synchronous motor (PMSM). Ref.[19] combined convolution neural network (CNN) and short-time Fourier transform(SFT) to realize motor fault diagnosis. In order to solve the problem of fault feature extraction is not deep enough and fault diagnosis accuracy is low, a new fault diagnosis method for permanent magnet synchronous motor(PMSM) combining non-linear spectrum based on generalized frequency response function (GFRF) and convolution neural network(CNN) is proposed. In this method, CNN network has strong ability of feature extraction and classification, but the input network is required to be the form of image, of all nonlinear spectrum based on Volterra kernel, only the second-order GFRF spectrum can meet the input requirement of CNN. In addition, the second-order GFRF spectrum is essentially two dimensional, which does not need to generate two dimensional data through the truncation and stacking of one dimensional data, thus it can avoid the loss of nonlinear characteristics[20]-[21]. Therefore, the second-order GFRF spectrum selected in this paper as CNN input retains the nonlinear characteristics of the data to the greatest extent. At the same time, according to the characteristics of GFRF spectrum, CNN network is designed, in which the convolution layer and the pooling layer alternately appear, and the learning rate of gradient descent is used to train the network to improve the accuracy of network output.

The rest of this paper is organized as follows: Section II will present the theory of GFRF spectrum and CNN; In Section Ⅲ, the process of the proposed method will be shown. Section IV will show the result of experiments and discuss the reasons why different methods produce vary recognition rates. Finally, a concise conclusion of this work will be presented in Section Ⅴ.

## 2. Methodology

### 2.1 The definition of GFRF spectrum

The input and output relationship of continuous time-invariant nonlinear dynamic system can be expressed by Volterra series as shown in Eq (1)[18], [22]-[24]:

$$y_{n}\left(t\right)=\overset{N}{\underset{n=1}{\sum }}\int_{-\infty }^{\infty }\int_{-\infty }^{\infty }\cdots \int_{-\infty }^{\infty }h_{n}\left(\tau_{1},\tau_{2},\cdots ,\tau_{n}\right)\overset{n}{\underset{i=1}{\prod }}u\left(t-\tau_{i}\right)d\tau_{i}$$ (1)

where, *u*(*t*) is the input of system, *y*_*n*_(*t*) is the n-th output of system, *h*_*n*_(*τ*_1_,*τ*_2_,⋯,*τ*_*n*_) is the n-th Volterra time domain kernel, then we make a multi-dimension Fourier transformation for *h*_*n*_(*τ*_1_,*τ*_2_,⋯,*τ*_*n*_),which can get the result as Eq (2):

$$H\left(j\omega_{1},j\omega_{2},\cdots ,j\omega_{n}\right)=\int_{-\infty }^{\infty }\cdots \int_{-\infty }^{\infty }h_{n}\left(\tau_{1},\tau_{2},\cdots ,\tau_{n}\right)e^{-j\left(\omega_{1}\tau_{1}+\omega_{2}\tau_{2}+\cdots +\omega_{n}\tau_{n}\right)}\overset{n}{\underset{i=1}{\prod }}d\tau_{i}$$ (2)

As shown in Eq (2), H(*jω*_1_,*jω*_2_,⋯,*jω*_*n*_) is called the n-th Volterra frequency domain kernel or generalized frequency response function (GFRF). A large number of experiments show that the GFRF spectrum of the system varies greatly in different states. According to this conclusion, we can characterize the spectrum of GFRF as features for fault diagnosis.

### 2.2 The calculation of GFRF spectrum

At present, there are two methods for GFRF calculating: the recursive method and the identification method. The former method calculates the GFRF spectrum values of each order by recursive equation, which is suitable for the system with clear mathematical model; the latter method evaluates the GFRF spectrum values of each order by system identification based on the input and output of the system, which is not only suitable for the system with clear mathematical model, but also suitable for the complex system whose mathematical model is unclear, so the latter method has stronger applicability.

According to the GFRF definition, the output of Volterra model $\tilde{y}(k)$ can be expressed as Eq (3):

$$\tilde{y}\left(k\right)=h_{v}\left(k\right)U_{v}^{T}\left(k\right)$$ (3)

where, $U_{v}^{T}(k)$ is the input vector, *h*_*v*_(*k*) is the Volterra kernel vector. From the analysis above, it can be seen that the identification of GFRF is essentially a least squares problem, In order to calculate the second GFRF spectrum accurately,this paper adopted a variable step size least mean square (VSSLMS) adaptive algorithm, which is shown as Eq (4).

$$h_{v}\left(k+1\right)=h_{v}\left(k\right)+\mu_{k}e\left(k\right)U_{v}^{T}\left(k\right)$$ (4)

where, *e*(*k*) is the error between $\tilde{y}(k)$ and y(*k*),which equals $\tilde{y}(k)-y(k)$. $U_{v}^{T}(k)$ is input of system. *μ*_*k*_ is the step size, which can be expressed as Eq (5).

$$\mu_{k}=\{\begin{array}{c} \mu_{max},\;\mu_{k}\geq \mu_{max}\\ \mu_{min},\;\mu_{k}\leq \mu_{min}\\ \mu_{k},\;other \end{array}$$ (5)

where *μ*_*k*_ = *γμ*_*k*−1_+*λ*|*e*(*k*−1)|^2^,0<*γ*<1,*λ*>0; *μ*_*max*_ is the upper limit of *μ*_*k*_; *μ*_*min*_ is the lower limit of *μ*_*k*_.

**Fig 1:**
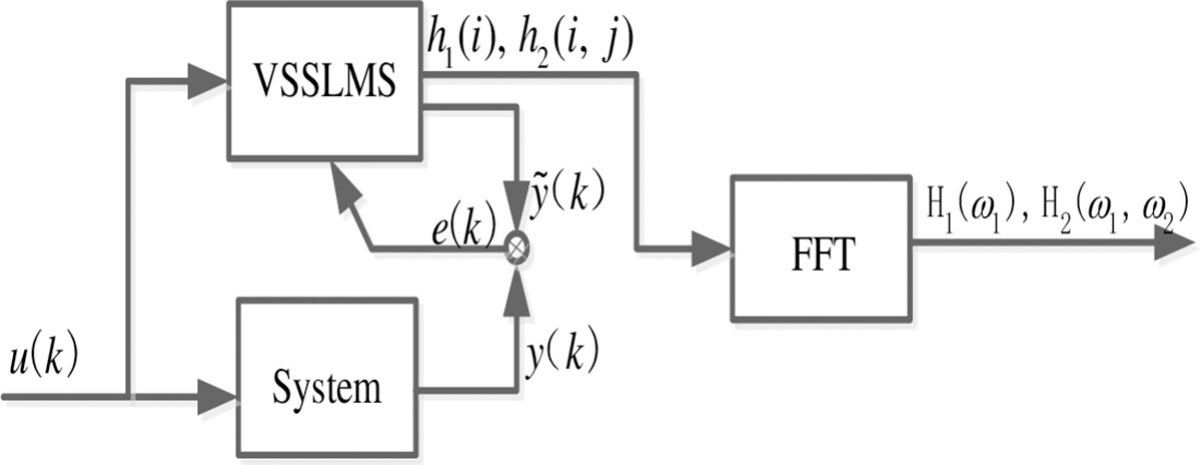
The process of GFRF calculation.

In Eq (5),the step size *μ*_*k*_ is decided by error *e*(*k*),namely, when the error *e*(*k*) is large, *μ*_*k*_ is also large, which will accelerate the convergence speed of algorithm; when the error *e*(*k*) is small, *μ*_*k*_ is also small, which will reduce the steady-state error of the algorithm. From the analysis above, it can be found that the adaptive identification algorithm as proposed can take into account both convergence speed and estimation error. The specific identification process is shown as Fig 1.

The specific steps of VSSLMS adaptive algorithm are as follows:

**Step 1**: Parameters initialization, letting *h*_*v*_(*k*) = [0,0,…,0]^*T*^, *μ*_*k*_ = *μ*_*max*_;

**Step 2:** Collecting the *k* input data $U_{v}^{T}(k)$ and *k* output data *y*(*k*);

**Step 3:** Calculating the estimated output $\tilde{y}(k)$ by (3),then calculating the error *e*(*k*), $e(k)=\tilde{y}(k)-y(k)$; if |*e*(*k*)|≪*ε*, the identification is over; else,it jump to step 4.

**Step 4:** Calculating the next Volterra series *h*_*v*_(*k*+1) by (3).

**Step 5:** Calculating the step size *μ*_*k*+1_, making *k* = *k*+1,and then jump to Step 3.

**Step 6:** when the algorithm is over, N×N Fourier Transform is performed on *h*_*v*_(*k*),and the second-order GFRF spectrum is obtained.

### 2.3 The theory of convolutional neural network

**Fig 2:**
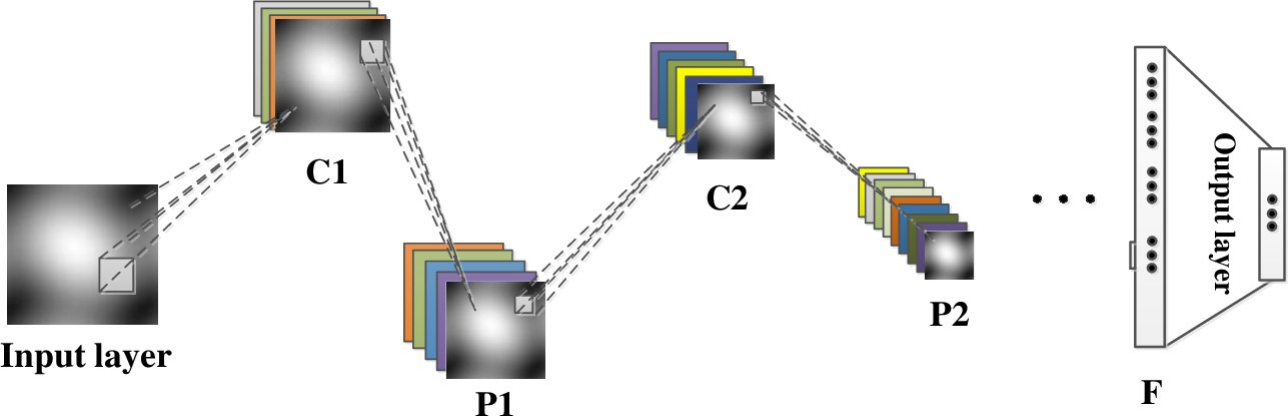
Convolutional neural network structure.

Convolutional neural network (CNN) is a kind of feedforward neural network, which can learn and extract information from data layer by layer, and also can reveal the essential characteristics of the system hidden in data. It is widely used in the field of image processing [25]-[27]. The network is usually composed of convolution layer(C), pooling layer(P) and full connection layer(F). In order to dig out the hidden characteristics of data, convolution layer and pooling layer often alternately appear. The structure of the network is shown as Fig 2.

### 2.3.1 Forward propagation

The forward propagation process of CNN network mainly involves convolution layer, pooling layer and full connection layer. The neurons between different layers are connected in the form of weights, which are important parameters of CNN, because they directly determine the mapping relationship of feature space.

Multiple convolution kernels are adopted in convolution layer to convolute the input images. After adding offset, some new features can be obtained by activation function. The mathematical expression of the convolution process can be shown as Eq (6):

$$X_{j}^{l}=f(\underset{i\in M_{j}}{\sum }X_{i}^{l-1}\cdot \omega_{ij}^{l}+b_{j}^{l})$$ (6)

where, $X_{j}^{l}$ is the *j*-th element in layer *l*; *M*_*j*_ is the set of input feature graphs needed to output the number *j* feature graph; $\omega_{ij}^{l}$ is the weight matrix of the corresponding convolution kernel. $b_{j}^{l}$ is the bias term; *f*(∙)is the activation function, and the ReLu function is commonly adopted, which is shown as Eq (7):

f(x)=max[0,x] (7)

The pooling layer is also called the lower sampling layer, which mainly reduces the dimension of the output feature map from the convolution layer. The pooling methods including maximum pooling, average pooling and random pooling are commonly used. Because the pooling layer only carries out dimension reduction operations and no parameters are involved in the calculation, it does not need to update the weights; the full connection layer mainly expands all the feature maps to form one dimensional feature vectors, and after weighted summation, it realizes the classification of feature types through the Softmax activation function.

### 2.3.2 Back propagation

Back propagation is used to adjust the network weight to minimize the error cost function, so as to minimize the error between the predicted results and the actual results in forward propagation to establish the mapping from the feature space to the fault space. There are many common cost functions. This paper chooses variance cost function, which is shown as Eq (8):

$$E=\frac{1}{2}\overset{n}{\underset{k=1}{\sum }}{\left(t_{k}^{n}-y_{k}^{n}\right)}^{2}=\frac{1}{2}\|t^{n}-y^{n}{\|}^{2}$$ (8)

where, *n* is the number of failure samples; *t*^*n*^ is the test value of the n-th sample; *y*^*n*^ is the true value of the n-th sample. In the process of reverse propagation, in order to obtain a series of convolution kernels, the gradient descent method is usually used to iterate continuously to adjust the adaptive parameters of the network. At the same time, the residual error of each layer is reduced to the minimum through learning, and finally the updated convolution kernels are obtained.

$$\omega_{ij}^{l}=\omega_{ij}^{l}-\delta \frac{\partial E}{\partial \omega_{ij}^{l}}$$ (9)

$$b_{j}^{l}=b_{j}^{l}-\delta \frac{\partial E}{\partial b_{j}^{l}}$$ (10)

As shown in Eqs (9)–(10), δ is a learning rate parameter, which is used to control the update step of the value. If δ is too large, the network will fall into local optimum. If δ is too small, the training time of the network will increase

## 3 Fault diagnosis method of permanent magnet synchronous motor based on GFRF spectrum and CNN

**Fig 3:**
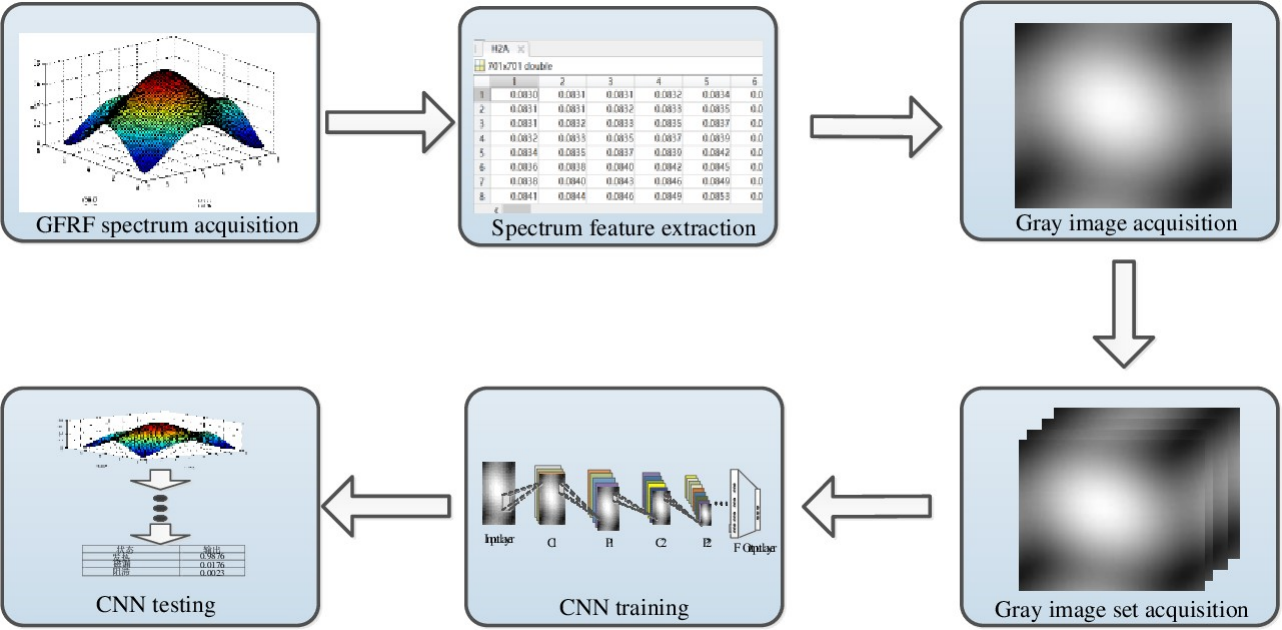
The flow of fault diagnosis based on GFRF spectrum +CNN.

In this paper, the fault diagnosis method based on GFRF spectrum + CNN can integrate fault feature extraction and pattern recognition to realize intelligent fault diagnosis of the system. The diagnosis process is shown as Fig 3.

The specific steps are as follows:

1. Obtaining the second-order GFRF spectrum of normal state and fault state of the system, and normalizing the spectrum value;
2. The normalized spectrum amplitudes were treated as image gray values, which were converted into two-dimension gray images.
3. A large number of gray image sets were obtained by repeated experiments N times, which were divided into training samples and test samples.
4. Initialize the convolution neural network and set parameters such as iteration times and learning rate.
5. The training samples were fed into CNN network in batches, the predicted values of fault types were obtained by forward propagation, and the errors were calculated with the actual values.
6. The error calculated in step (5) was back-propagated, and the weight parameters of each layer were updated layer by layer to obtain the ideal CNN network.
7. The test samples were put into CNN network to realize the output of fault types of test samples.

## 4 Experimental verification

### 4.1 Data acquisitions

In this paper, the permanent magnet synchronous motor (PMSM) used in industrial robot is taken as the research object for fault diagnosis. According to the mathematical model of PMSM in Ref. [28], it can be expressed as Eq (11):

$$\begin{array}{c} \frac{di_{d}}{dt}=-\frac{r}{L}i_{d}+p\omega_{r}i_{q}+\frac{1}{L}u_{d}\\ \frac{di_{q}}{dt}=-p\omega_{r}i_{d}-\frac{r}{L}i_{q}-\frac{\phi_{g}p}{L}\omega_{r}+\frac{1}{L}u_{q}\\ \frac{d\omega_{r}}{dt}=\frac{1.5\phi_{g}p}{J}i_{q}-\frac{B}{J}\omega_{r}+\frac{T_{l}}{J} \end{array}$$ (11)

As shown in Eq (11), *i*_*d*_ and *i*_*q*_ are armature currents of stator winding on *d* axis and *q* axis respectively; *u*_*d*_ and *u*_*q*_ are armature voltage of stator winding on d axis and q axis respectively. For ease of analysis, this paper assumes *u*_*d*_ = 0; L is stator winding inductance; ϕ_*g*_ is flux linkage produced by permanent magnet; *r* is stator winding resistance; J is rotor moment of inertia; T_*l*_ is load torque; p is the number of pole pair; B is rotor damping coefficient; ω_*r*_ is rotor angular velocity. In (10), *i*_*d*_ is input and ω_*r*_ is output of the system, respectively, the nonlinear differential model can be expressed as Eq (12).

$$i_{d}^{\prime }+\frac{r}{L}i_{d}-\frac{2J}{3\phi_{g}}\omega_{r}\omega_{r}^{\prime }-\frac{2B}{3\phi_{g}}\omega_{r}^{2}-\frac{2T_{l}}{3\phi_{g}}\omega_{r}=0$$ (12)

During the long-term operation of the equipment, the motor often suffers from fever, poor lubrication and magnetic leakage of the rotor. These fault states are represented by parameter r increasing, parameter B increasing and parameter ϕ_*g*_ decreasing, respectively. Under normal conditions, the parameters of a certain type of permanent magnet synchronous motor (PMSM)are as follows:

$$\begin{array}{c} r\in [5.0,5.5]\Omega ,T_{l}=3N∙m,L=0.005H,\\ \phi_{g}=0.186Wb,J=1.5\times 10^{-6}kg∙m^{2},\\ B\in [2.0\times 10^{-5},3.2\times 10^{-5}]N∙m∙s/rad \end{array}$$

when fever failure occurs, the parameters are as follows:

$$\begin{array}{c} r>5.6\Omega ,T_{l}=3N∙m,L=0.005H,\\ \phi_{g}=0.186Wb,J=1.5\times 10^{-6}kg∙m^{2},\\ B\in [2.0\times 10^{-5},3.2\times 10^{-5}]N∙m∙s/rad \end{array}$$

when poor lubrication of rotor failure occurs, the parameters are as follows:

$$\begin{array}{c} r\in [5.0,5.5]\Omega ,T_{l}=3N∙m,L=0.005H,\\ \phi_{g}=0.186Wb,J=1.5\times 10^{-6}kg∙m^{2},\\ B>3.2\times 10^{-5}N∙m∙s/rad \end{array}$$

when magnetic leakage of rotor failure occurs, the parameters are as follows:

$$\begin{array}{c} r\in [5.0,5.5]\Omega ,T_{l}=3N∙m,L=0.005H,\\ \phi_{g}\in [0,0.186]Wb,J=1.5\times 10^{-6}kg∙m^{2},\\ B\in [2.0\times 10^{-5},3.2\times 10^{-5}]N∙m∙s/rad \end{array}$$

**Fig 4:**
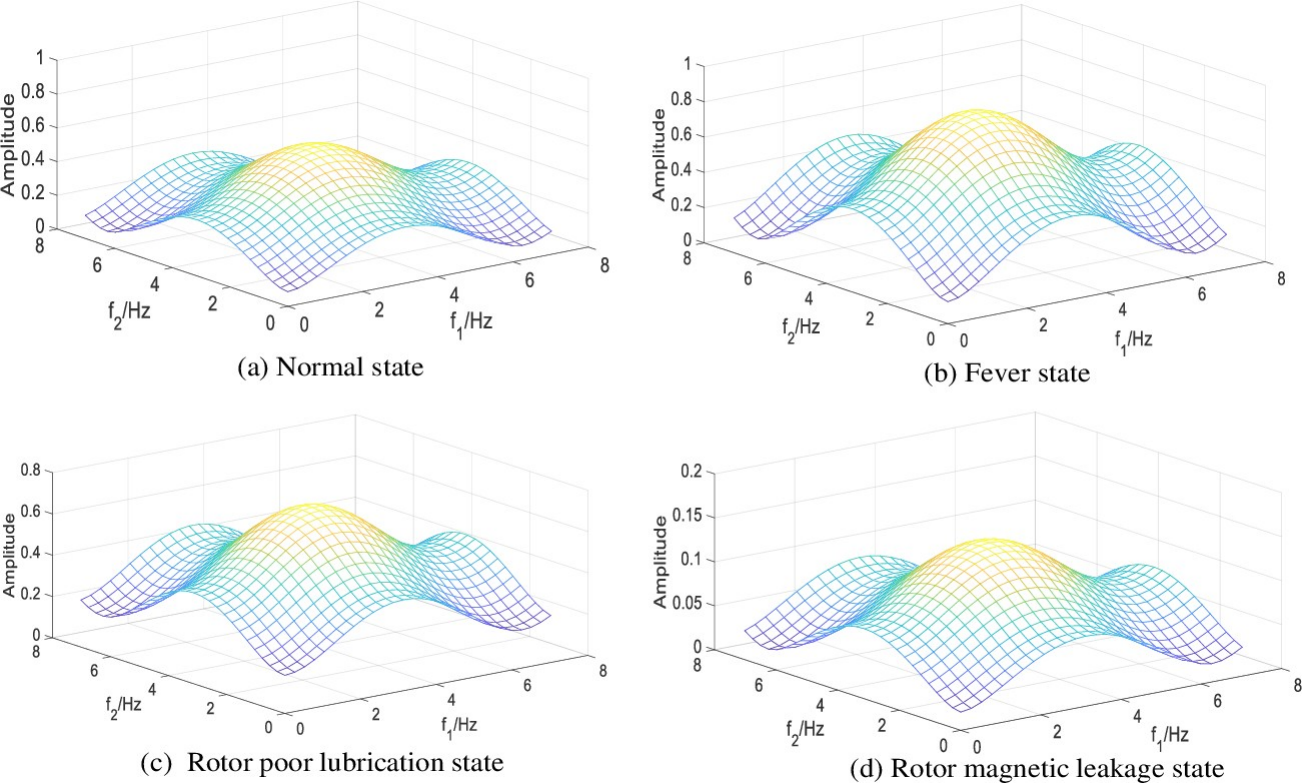
Second-order GFRF spectrum in different states under working condition 1.

Adopting the identification algorithm of VSSLMS proposed in this paper, the second-order GFRF spectrums of normal state and three failure states are shown as (Fig 4A–4D) under the conditions as Table 1.

**Table 1:** Corresponding parameters of different states under working condition 1.

State	r (Ω)	B (N·m·s/rad)	*ϕ*_g_ (Wb*)*
Normal	5	3×10^−5^	0.186
Fever	6	3×10^−5^	0.186
Rotor poor lubrication	5	6.4×10^−5^	0.186
Rotor magnetic leakage	5	3×10^−5^	0.091

The peak values of the second-order GFRF spectrum under working condition 1 are shown as Table 2.

**Table 2:** Peak distribution of second-order GFRF spectrum in different states.

State	Second-order GFRF spectrum value	
	Maximum	Minimum
Normal	0.6279	0.0865
Fever	0.8769	0.1245
Rotor poor lubrication	0.7465	0.1845
Rotor magnetic leakage	0.1503	0.0207

**Fig 5:**
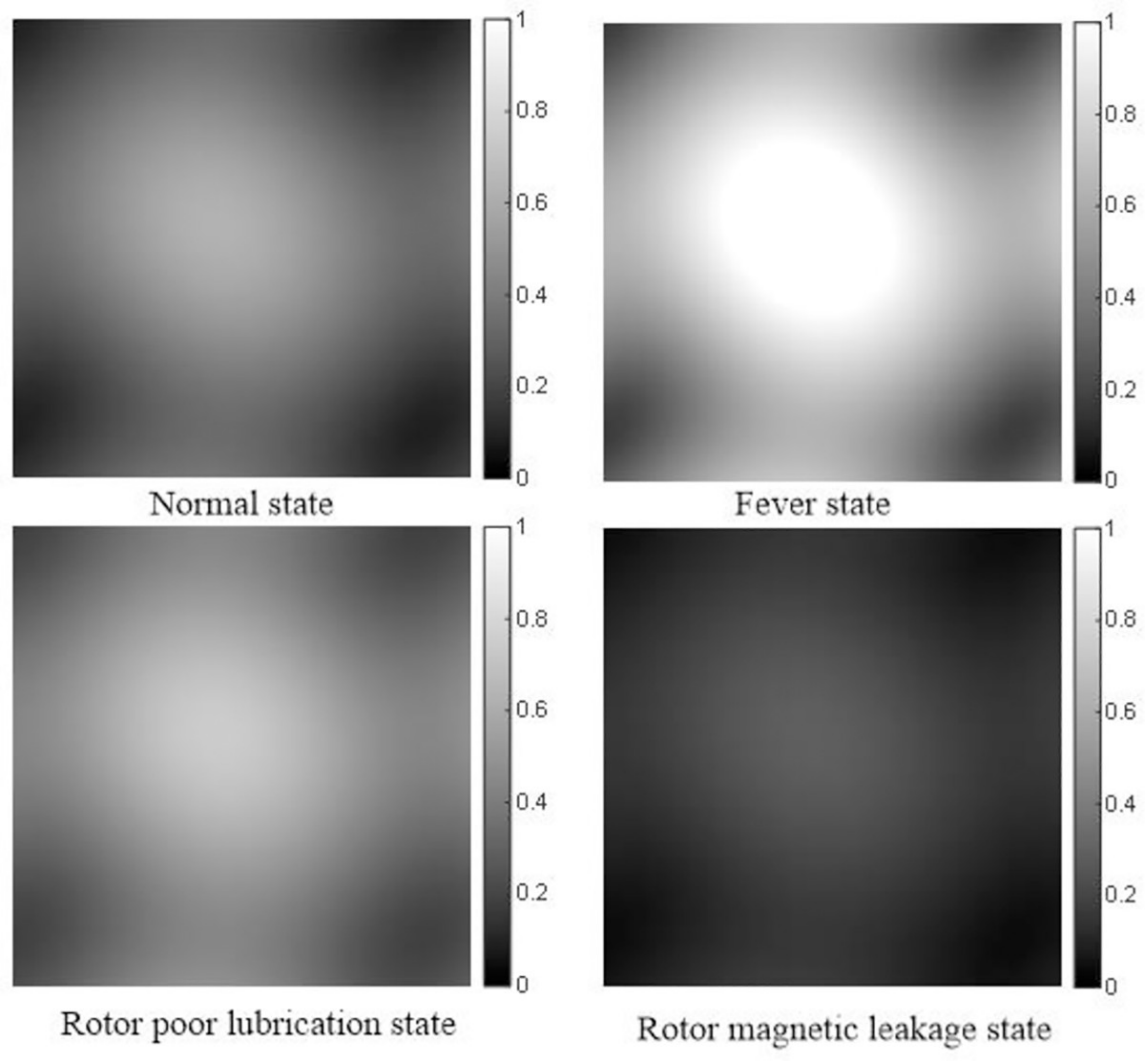
Gray images of GFRF spectrum.

From Fig 4 and Table 2, it can be seen that the second-order GFRF spectrum values vary greatly in different states, so the fault can be characterized by the second-order GFRF spectrum. In order to satisfy the requirement of CNN input type, the spectrum values are treated as gray values of image, so that the spectrum of different states can be converted into two-dimension gray images, which are shown as Fig 5. Each state is repeated 30,000 times, so 30,000 two-dimension gray image sets are obtained. 80% of images are trained and 20% of images are tested in CNN.

### 4.2 Experiment results and analysis

The CNN network constructed in this paper consists of three convolution layers (C1, C2, C3), two pooling layers (P1, P2), two full connection layers (F1, F2) and one Softmax output layer (O1). Three convolution layers and two pooling layers appear alternately. The number of convolution kernels in convolution layers are 36, 66 and 240. the size of convolution kernels is 5 *5, and the sliding step is 1; the activation function is adopted ReLU function; the average pooling operation is used in the pooling layer, and the pooling area is 2 *2. The learning rate is uniformly changed, namely, the initial learning rate is set to 0.01, and the value is reduced to 1/2 of the original value every 500 steps; the number of F1 and F2 in the full connection layer are 86 and 4, respectively. The input gray image size is 28 *28 and the batch equals 1000. The laboratory platform is configured as follows: Linux 18.04 operating system, GPU model is GTX1060, and the running environment is Python 3.7 + tensorflow 1.6. In order to prove the superiority of the proposed method, the following comparative experiments are carried out from the following aspects.

### 4.2.1 Comparison with the output signal+CNN methods

**Fig 6:**
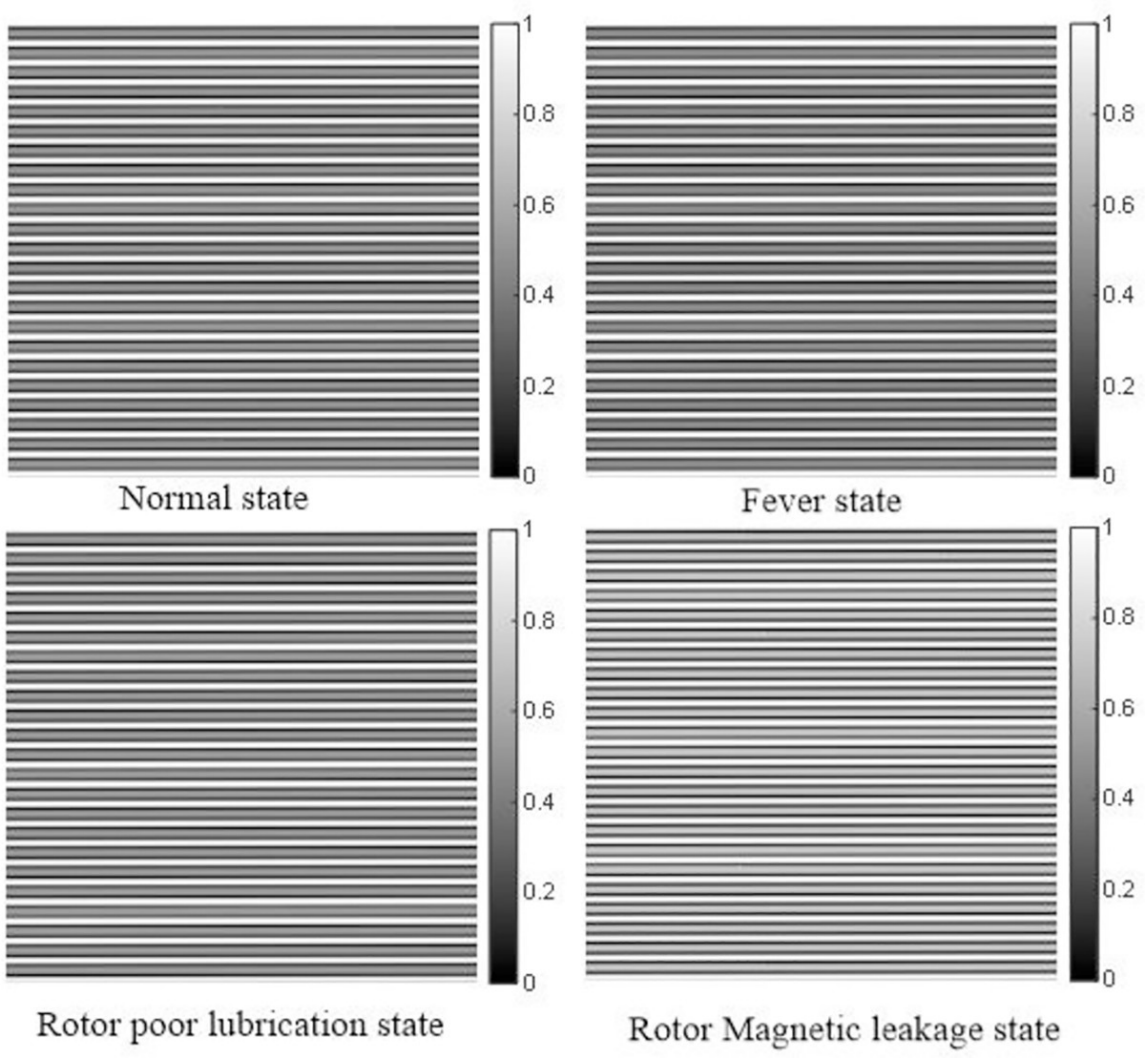
Gray images of time domain of output.

**Fig 7:**
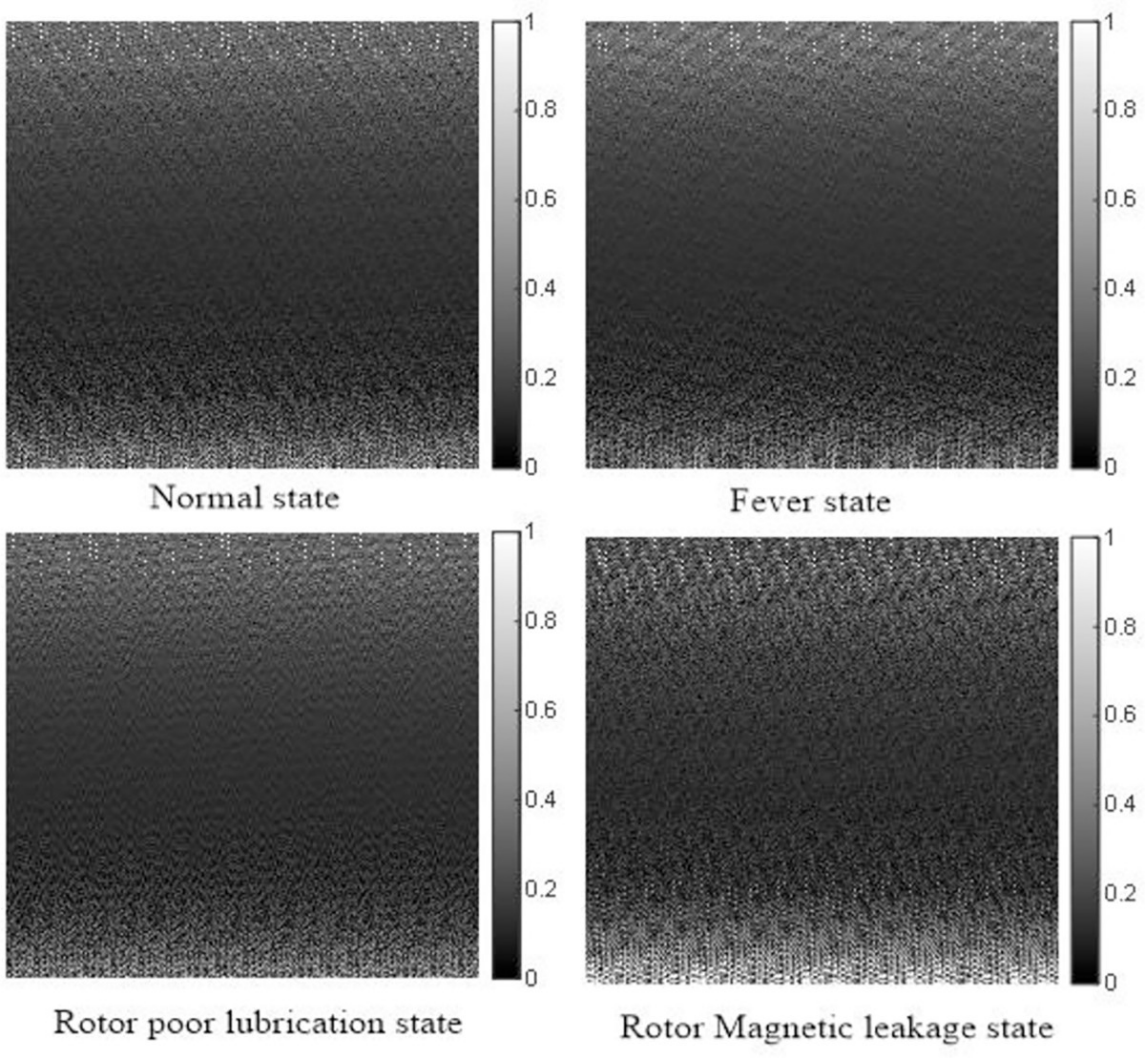
Gray images of frequency domain of output.

Most of data in current fault diagnosis research are collected from system output, such as current or vibration signal [2]-[6]. In order to verify the reliability of different data sets and their impacts on diagnosis results, the data set of this paper is GFRF spectrum estimated by VSSLMS, while the comparative test data sets are as follows: collecting the time domain signal(TS) of rotor speed *ω*_*r*_,and the frequency domain signal (FS) of *ω*_*r*_ generated by Fourier transform. Meanwhile, the two kinds of one-dimension signal are intercepted and stacked to generate gray images, which are shown as Figs 6 and 7.

Putting the two types of gray images into the same CNN network of this paper for diagnosis. The diagnosis results are shown as Table 3.

**Table 3:** Recognition rates of different fault diagnosis methods.

Method	State	Samples	Misjudgments	Recognition rate	Average rate
GFRF+CNN	Normal	5941	0	100.00%	98.75%
	Fever	5946	298	94.99%	
	Rotor poor lubrication	5996	0	100.00%	
	Rotor magnetic leakage	6102	0	100.00%	
FS+CNN	Normal	5990	113	98.11%	83.65%
	Fever	5952	864	85.48%	
	Rotor poor lubrication	5989	3054	50.99%	
	Rotor magnetic leakage	6094	0	100.00%	
TS+CNN	Normal	5922	651	89.01%	78.08%
	Fever	6001	522	91.30%	
	Rotor poor lubrication	5909	4018	32.00%	
	Rotor magnetic leakage	6062	0	100.00%	

From the Table 3, it can be seen that the average accuracy of GFRF spectrum + CNN method is 98.75%, the average accuracy of FS + CNN method is 83.65% and the average accuracy of TS + CNN method is 78.08%. The reason for this result is that the fault representation based on GFRF spectrum is designed from the whole system, which contains most information of the non-linear characteristics of the system. The distinctions between different faults are obvious, which can be seen from Fig 5, so the diagnosis accuracy is high. The output time-domain or frequency-domain signal only contain the local information of the system, which cannot represent the whole non-linear characteristics of the system. Meanwhile, unlike the second-order GFRF spectrum, the output data need to be converted into two-dimension gray image by interception and superposition. This operation may lead to the loss of non-linear characteristics. Therefore, the distinctions between different faults are not obvious, which can be seen from Fig 6 and Fig 7. It is worthy to be noticed that compared with Fig 6 and Fig 7, it is found that although the distinctions of output signal gray images in different states are not obvious, it is still discernible, gray images formed by output time domain signal cannot be directly distinguished by eyes. It is consistent with the conclusion that the information contained in frequency domain signal is more than that contained in time domain signal. Therefore, the accuracy of fault diagnosis using output time-domain signal to represent fault features is the lowest.

### 4.2.2. Comparison with other learning networks

In order to compare the fault diagnosis accuracy of different learning networks, three other deep learning networks, such as stack auto-encoder(SAE) network in Ref. [18],[29], deep belief neural (DBN)network in Ref.[30] and recurrent neural network(RNN) in Ref.[31] are adopted to diagnose PMSM faults. The accuracy of four methods are shown as Table 4.

**Table 4:** The fault diagnosis accuracy of different methods.

Method	Samples	Average accuracy rate
CNN	4×6000	98.75%
SAE	4×6000	96.33%
DBN	4×6000	97.69%
RNN	4×6000	94.14%

From Table 4, it can be seen that adopting deep learning network to diagnose the fault of PMSM can achieve high diagnosis accuracy, it is because that the deep learning network can extract fault features layer by layer, which can accurately reflect the changes of state. However, for PMSM system, the accuracy of fault diagnosis using CNN method is the highest.

### 4.2.3 Comparisons with traditional intelligent diagnosis methods

**Fig 8:**
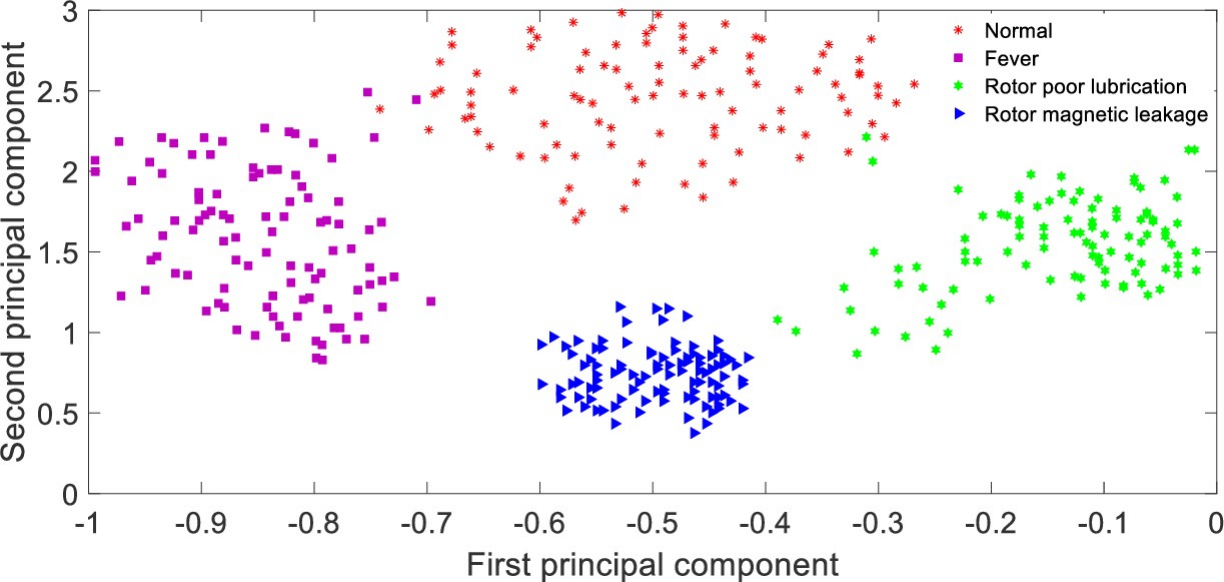
Visualization of principal components based on CNN feature extraction.

**Fig 9:**
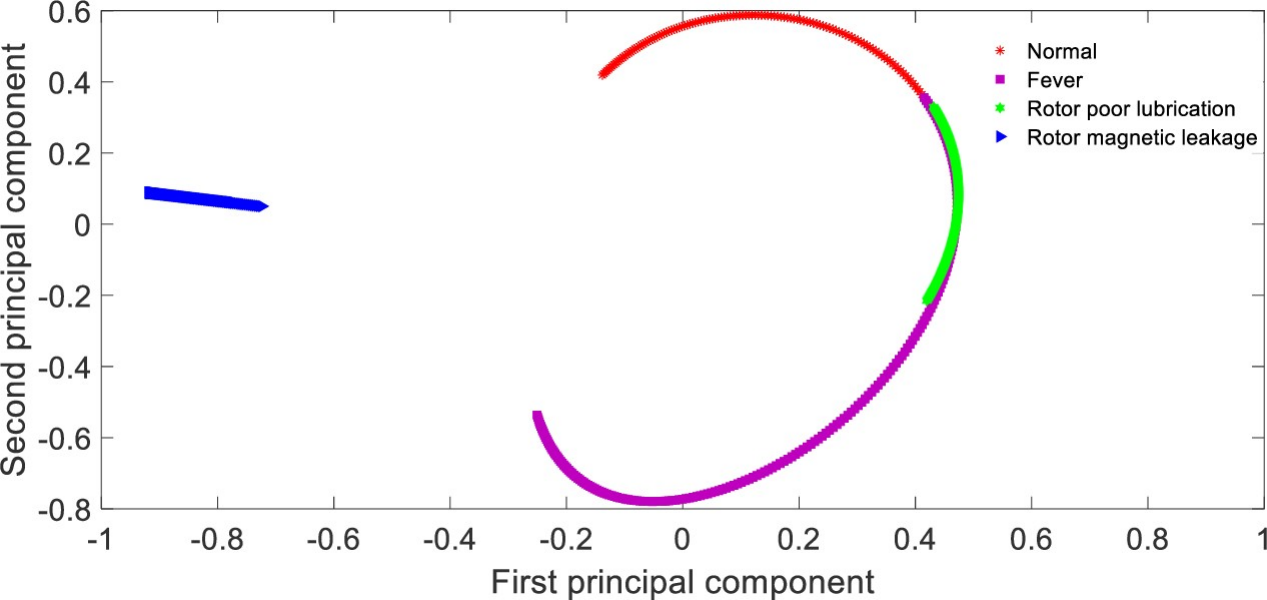
Visualization of principal components based on PCA feature extraction.

**Fig 10:**
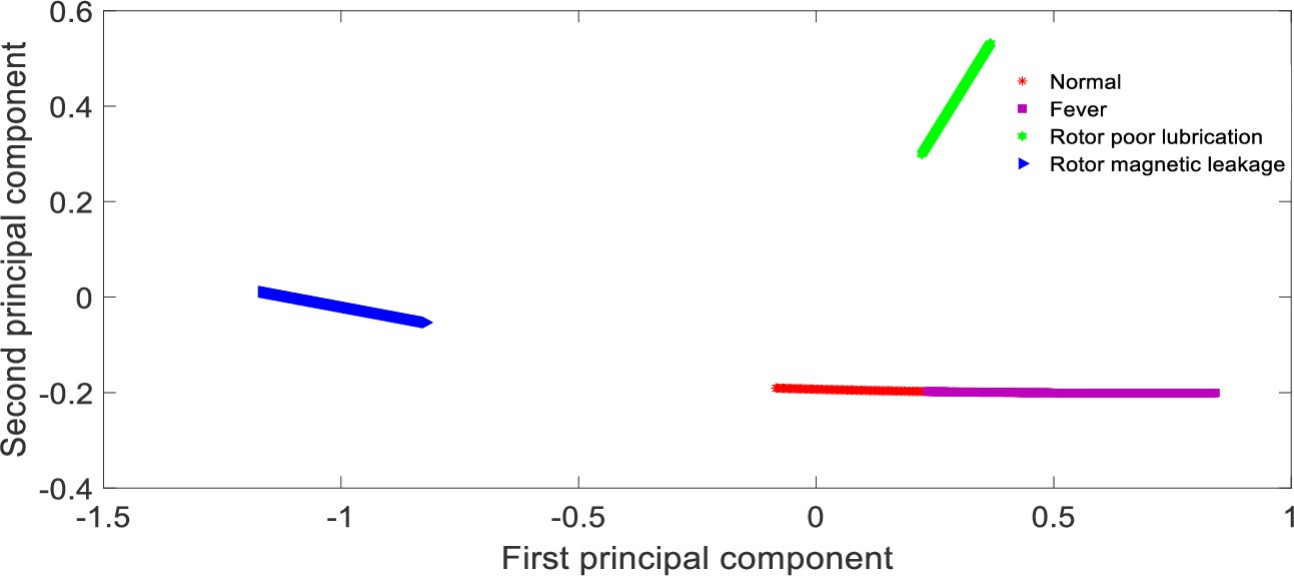
Visualization of principal components based on KPCA feature Extraction.

Feature extraction and pattern recognition are two processes of equipment fault diagnosis. At present, PCA in Ref. [32], KPCA in Ref. [33], t-SNE in Refs.[34]-[35] are the main methods of feature extraction, and SVM in Ref. [36] is the main method of fault recognition. Therefore, this paper compares the method based on GFRF spectrum + CNN with PCA + SVM, KPCA + SVM and t-SNE +SVM. The data sources of the latter methods are as follows: 800 points of second-order GFRF spectrum are collected randomly (mainly near the peak value) to form a set of 800-dimension vector, 200*800 data sets are obtained by 200 times experiments, 80% and 20% of which are treated as training sets and testing sets respectively. Gauss kernel function is adopted as KPCA kernel function, and the parameter of kernel function *σ*^2^ equals 4000, the cumulative contribution rate of principal components is set to 90%. The diagnosis accuracy of these methods are shown as Table 5. Meanwhile, the principal component analysis method is used to extract first two important components of fault features from three methods, and the visualization of first two principal components are shown as Figs 8–10.

**Table 5:** Accuracy rates of different fault diagnosis methods.

Method	State	Samples	Misjudgments	Accuracy rate	Average rate
**GFRF+CNN**	Normal	5941	0	100.00%	98.75%
	Fever	5946	298	94.99%	
	Rotor poor lubrication	5996	0	100.00%	
	Rotor magnetic leakage	6102	0	100.00%	
**KPCA+SVM**	Normal	40	40	0.00%	63.13%
	Fever	40	0	100.00%	
	Rotor poor lubrication	40	19	52.50%	
	Rotor magnetic leakage	40	0	100.00%	
**PCA+SVM**	Normal	40	40	0.00%	26.88%
	Fever	40	0	100.00%	
	Rotor poor lubrication	40	40	0.00%	
	Rotor magnetic leakage	40	37	7.50%	
**t-SNE+SVM**	Normal	40	19	52.50%	82.50%
	Fever	40	0	100.00%	
	Rotor poor lubrication	40	8	80.00%	
	Rotor magnetic leakage	40	1	97.50%	

**Fig 11:**
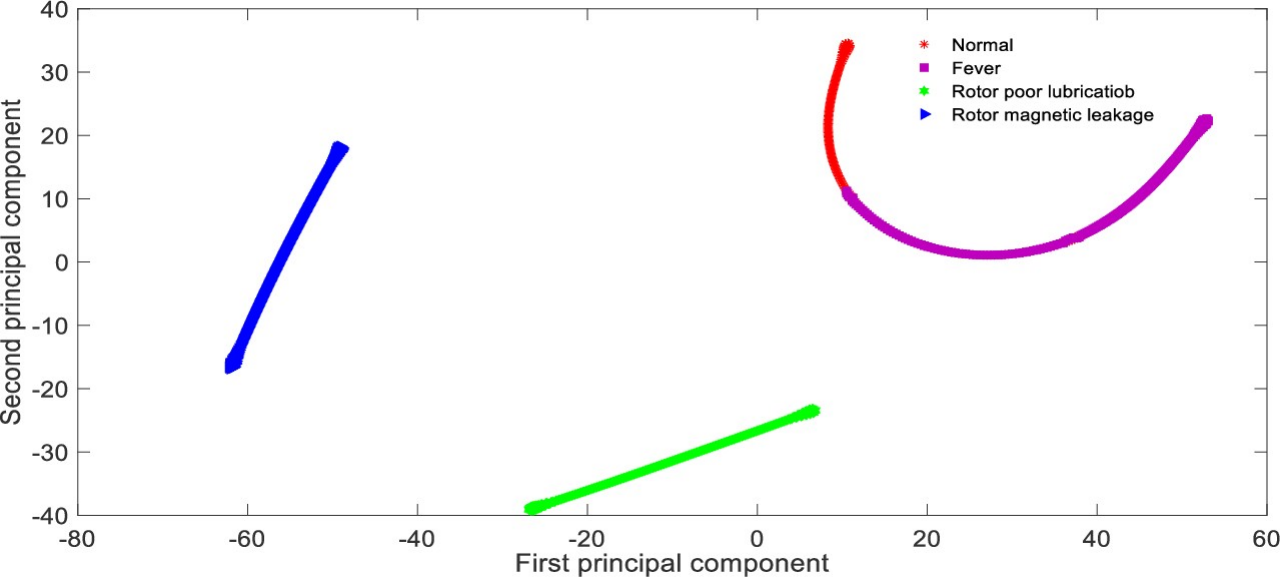
Visualization of principal components based on t-SNE feature extraction.

From Table 5, it can be seen that the recognition rate of the proposed method is 98.75%, the recognition rate of KPCA + SVM method is 63.13%, the recognition rate of PCA + SVM method is only 26.88%, and the recognition rate of t-SNE + SVM method is 82.50%. The reason for this result is that in this paper, CNN with multi-layer is adopted to extract fault characteristics layer by layer, and the network parameters can be adjusted by back propagation, which has strong self-adaptability. Therefore, the features distinction between different states are obvious, which can be seen from Fig 8; PCA+SVM adopts linear mapping function to compress high-dimension data, which is obviously inappropriate for a non-linear system. After dimension reduction, the state features contained in the data overlap with each other, which is shown as Fig 9; KPCA + SVM adopts non-linear mapping function of Gauss to improve the features discrimination between different faults, the features distinction is shown as Fig 10; Compared with the Gauss distribution in KPCA, the t-distribution adopted in t-SNE method is less affected by the outliers, the fitting results are more reasonable, and the overall characteristics of the data are better captured, the features distinction is shown as Fig 11.

## 5 Conclusions

A new method of fault diagnosis for permanent magnet synchronous motor(PMSM) is proposed. It combines the nonlinear frequency spectrum based on GFRF and convolution neural network(CNN) to improve the diagnosis accuracy. Nonlinear frequency spectrum based on GFRF is utilized to characterize the fault information, the CNN is utilized to extract fault features from GFRF spectrum, experimental results verified the effectiveness of the proposed method.

The main achievements can be summarized as follows:

1. Compared with the method of fault diagnosis using output signal as fault feature, nonlinear spectrum based on GFRF can fully represent the state information of the system, which has higher diagnosis accuracy rate.
2. Compared with other deep learning network, CNN has highest ability of feature extraction and classification for PMSM fault diagnosis.
3. Compared with traditional diagnosis method PCA, KPCA and t-SNE, convolutional neural network has obvious advantages in fault diagnosis accuracy with its powerful data mining and strong fault feature extraction capabilities.

Although the proposed method has achieved excellent results for fault diagnosis of PMSM, it will be a future work to test the versatility of the proposed method by diagnosing other non-linear systems.

## Supporting information

S1 Fig The process of GFRF Calculation. (DOCX)

S2 Fig Convolutional neural network structure. (DOCX)

S3 Fig The flow of fault diagnosis based on GFRF Spectrum +CNN. (DOCX)

S4 Fig Second-order GFRF spectrum in different states under working condition 1. (DOCX)

S5 Fig Gray images of GFRF spectrum. (DOCX)

S6 Fig Gray images of time domain of output. (DOCX)

S7 Fig Gray images of frequency domain of output. (DOCX)

S8 Fig Visualization of principal components based on CNN feature extraction. (DOCX)

S9 Fig Visualization of principal components based on PCA feature extraction. (DOCX)

S10 Fig Visualization of principal components based on KPCA feature extraction. (DOCX)

S11 Fig Visualization of principal components based on t-SNE feature extraction. (DOCX)

S1 Table Corresponding parameters of different states under working condition 1. (DOCX)

S2 Table Peak distribution of second-order GFRF spectrum in different states. (DOCX)

S3 Table Recognition rates of different fault diagnosis methods. (DOCX)

S4 Table The fault diagnosis accuracy of different methods. (DOCX)

S5 Table Accuracy rates of different fault diagnosis methods. (DOCX)
