# Supplementary material for: A novel method of combining generalized frequency response function and convolutional neural network for complex system fault diagnosis
Source: PLoS One. 2020 Feb 4;15(2):e0228324. doi: 10.1371/journal.pone.0228324 (PMC6999895; doi:10.1371/journal.pone.0228324)
Supplement: S3 Fig — (DOCX) [file pone.0228324.s003.docx]

**S3 Fig. The flow of fault diagnosis based on GFRF Spectrum +CNN**
