# Supplementary figures and images for: A novel method of combining generalized frequency response function and convolutional neural network for complex system fault diagnosis
Source: PLoS One. 2020 Feb 4;15(2):e0228324. doi: 10.1371/journal.pone.0228324 (PMC6999895; doi:10.1371/journal.pone.0228324)

**S5 Fig. Gray images of GFRF spectrum**


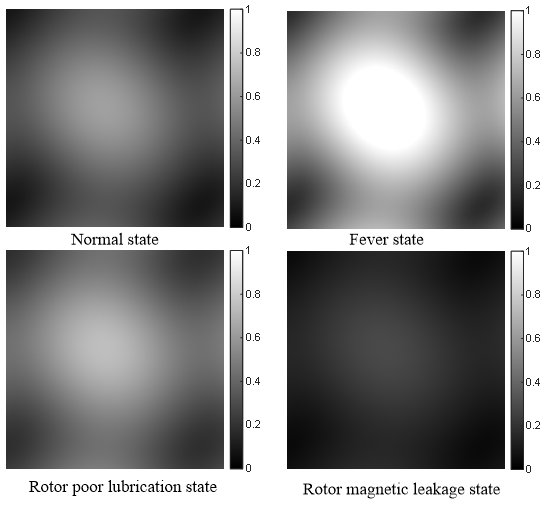

Supplement: S5 Fig — (DOCX) [file pone.0228324.s005.docx]

**S6 Fig. Gray images of time domain of output**


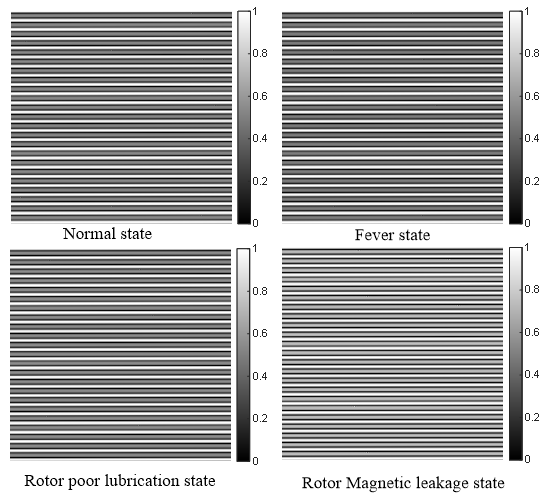

Supplement: S6 Fig — (DOCX) [file pone.0228324.s006.docx]

**S7 Fig. Gray images of frequency domain of output**


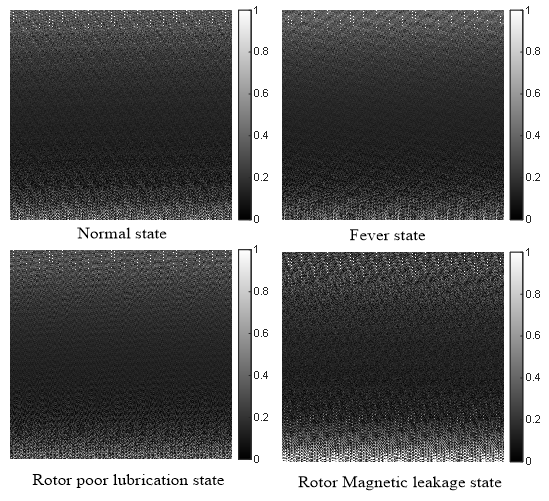

Supplement: S7 Fig — (DOCX) [file pone.0228324.s007.docx]
