# Supplementary material for: A novel method of combining generalized frequency response function and convolutional neural network for complex system fault diagnosis
Source: PLoS One. 2020 Feb 4;15(2):e0228324. doi: 10.1371/journal.pone.0228324 (PMC6999895; doi:10.1371/journal.pone.0228324)
Supplement: S8 Fig — (DOCX) [file pone.0228324.s008.docx]

**S8 Fig. Visualization of principal components based on CNN feature extraction**
