# Supplementary material for: A novel method of combining generalized frequency response function and convolutional neural network for complex system fault diagnosis
Source: PLoS One. 2020 Feb 4;15(2):e0228324. doi: 10.1371/journal.pone.0228324 (PMC6999895; doi:10.1371/journal.pone.0228324)
Supplement: S1 Table — (DOCX) [file pone.0228324.s012.docx]

**S1 Table. Corresponding parameters of different states under working condition 1**

| **State** | **$\text{r}$(Ω)** | | | **$\text{B}$（N·m·s/rad）** | (**Wb*)*** |
| --- | --- | --- | --- | --- | --- |
| Normal | | 5 | | 3×10^-5^ | 0.186 |
| Fever | | 6 | | 3×10^-5^ | 0.186 |
| Rotor poor lubrication | | | 5 | 6.4×10^-5^ | 0.186 |
| Rotor magnetic leakage | | | 5 | 3×10^-5^ | 0.091 |
