# Supplementary material for: A novel method of combining generalized frequency response function and convolutional neural network for complex system fault diagnosis
Source: PLoS One. 2020 Feb 4;15(2):e0228324. doi: 10.1371/journal.pone.0228324 (PMC6999895; doi:10.1371/journal.pone.0228324)
Supplement: S2 Table — (DOCX) [file pone.0228324.s013.docx]

**S2 Table. Peak distribution of second-order GFRF spectrum in different states**

| **State** | **Second-order GFRF spectrum value** | |
| --- | --- | --- |
|  | Maximum | Minimum |
| Normal | 0.6279 | 0.0865 |
| Fever | 0.8769 | 0.1245 |
| Rotor poor lubrication | 0.7465 | 0.1845 |
| Rotor magnetic leakage | 0.1503 | 0.0207 |
