# Supplementary material for: A novel method of combining generalized frequency response function and convolutional neural network for complex system fault diagnosis
Source: PLoS One. 2020 Feb 4;15(2):e0228324. doi: 10.1371/journal.pone.0228324 (PMC6999895; doi:10.1371/journal.pone.0228324)
Supplement: S3 Table — (DOCX) [file pone.0228324.s014.docx]

**S3 Table. Recognition rates of different fault diagnosis methods**

| **Method** | **State** | **Samples** | **misjudgments** | **Recognition rate** | **Average rate** |
| --- | --- | --- | --- | --- | --- |
| GFRF+CNN | Normal | 5941 | 0 | 100.00% | 98.75% |
|  | Fever | 5946 | 298 | 94.99% |  |
|  | Rotor poor lubrication | 5996 | 0 | 100.00% |  |
|  | Rotor magnetic leakage | 6102 | 0 | 100.00% |  |
| FS+CNN | Normal | 5990 | 113 | 98.11% | 83.65% |
|  | Fever | 5952 | 864 | 85.48% |  |
|  | Rotor poor lubrication | 5989 | 3054 | 50.99% |  |
|  | Rotor magnetic leakage | 6094 | 0 | 100.00% |  |
| TS+CNN | Normal | 5922 | 651 | 89.01% | 78.08% |
|  | Fever | 6001 | 522 | 91.30% |  |
|  | Rotor poor lubrication | 5909 | 4018 | 32.00% |  |
|  | Rotor magnetic leakage | 6062 | 0 | 100.00% |  |
