# Supplementary material for: A novel method of combining generalized frequency response function and convolutional neural network for complex system fault diagnosis
Source: PLoS One. 2020 Feb 4;15(2):e0228324. doi: 10.1371/journal.pone.0228324 (PMC6999895; doi:10.1371/journal.pone.0228324)
Supplement: S4 Table — (DOCX) [file pone.0228324.s015.docx]

**S4 Table. The fault diagnosis accuracy of different methods**

| **Method** | **Samples** | **Average accuracy rate** |
| --- | --- | --- |
| CNN | 4×6000 | 98.75% |
| SAE | 4×6000 | 96.33% |
| DBN | 4×6000 | 97.69% |
| RNN | 4×6000 | 94.14% |
