# Supplementary material for: A novel method of combining generalized frequency response function and convolutional neural network for complex system fault diagnosis
Source: PLoS One. 2020 Feb 4;15(2):e0228324. doi: 10.1371/journal.pone.0228324 (PMC6999895; doi:10.1371/journal.pone.0228324)
Supplement: S5 Table — (DOCX) [file pone.0228324.s016.docx]

**S5 Table. Accuracy rates of different fault diagnosis methods**

| **Method** | **State** | **Samples** | **Misjudgments** | **Accuracy rate** | **Average rate** |
| --- | --- | --- | --- | --- | --- |
| **GFRF+CNN** | Normal | 5941 | 0 | 100.00% | 98.75% |
|  | Fever | 5946 | 298 | 94.99% |  |
|  | Rotor poor lubrication | 5996 | 0 | 100.00% |  |
|  | Rotor magnetic leakage | 6102 | 0 | 100.00% |  |
| **KPCA+SVM** | Normal | 40 | 40 | 0.00% | 63.13% |
|  | Fever | 40 | 0 | 100.00% |  |
|  | Rotor poor lubrication | 40 | 19 | 52.50% |  |
|  | Rotor magnetic leakage | 40 | 0 | 100.00% |  |
| **PCA+SVM** | Normal | 40 | 40 | 0.00% | 26.88% |
|  | Fever | 40 | 0 | 100.00% |  |
|  | Rotor poor lubrication | 40 | 40 | 0.00% |  |
|  | Rotor magnetic leakage | 40 | 37 | 7.50% |  |
| **t-SNE+SVM** | Normal | 40 | 19 | 52.50% | 82.50% |
|  | Fever | 40 | 0 | 100.00% |  |
|  | Rotor poor lubrication | 40 | 8 | 80.00% |  |
|  | Rotor magnetic leakage | 40 | 1 | 97.50% |  |
